# Ethanolic Extract of *Orthosiphon stamineus* Improves Memory in Scopolamine-Induced Amnesia Model

**DOI:** 10.3389/fphar.2019.01216

**Published:** 2019-10-29

**Authors:** Thaarvena Retinasamy, Mohd Farooq Shaikh, Yatinesh Kumari, Iekhsan Othman

**Affiliations:** Neuropharmacology Research Laboratory, Jeffrey Cheah School of Medicine and Health Sciences, Monash University Malaysia, Bandar Sunway, Malaysia

**Keywords:** Alzheimer’s disease, *Orthosiphon stamineus*, scopolamine, animal model, learning and memory

## Abstract

Alzheimer’s disease (AD) is a chronic neurodegenerative brain disease which is characterized by impairment in cognitive functioning. *Orthosiphon stamineus* (OS) Benth. (Lamiaceae) is a medicinal plant found around Southeast Asia that has been employed as treatments for various diseases. OS extract contains many active compounds that have been shown to possess various pharmacological properties whereby *in vitro* studies have demonstrated neuroprotective as well as cholinesterase inhibitory effects. This study, therefore aimed at determining whether this Malaysian plant derived flavonoid can reverse scopolamine induced learning and memory dysfunction in the novel object recognition (NOR) test and the elevated plus maze (EPM) test. In the present study, rats were treated once daily with OS 50 mg/kg, 100 mg/kg, 200 mg/kg and donepezil 1 mg/kg *via* oral dosing and were given intraperitoneal (ip) injection of scopolamine 1 mg/kg daily to induce cognitive deficits. Rats were subjected to behavioral analysis to assess learning and memory functions and hippocampal tissues were extracted for gene expression and immunohistochemistry studies. All the three doses demonstrated improved scopolamine-induced impairment by showing shortened transfer latency as well as the higher inflexion ratio when compared to the negative control group. OS extract also exhibited memory-enhancing activity against chronic scopolamine-induced memory deficits in the long-term memory novel object recognition performance as indicated by an increase in the recognition index. OS extract was observed to have modulated the mRNA expression of CREB1, BDNF, and TRKB genes and pretreatment with OS extract were observed to have increased the immature neurons against hippocampal neurogenesis suppressed by scopolamine, which was confirmed by the DCX-positive stained cells. These research findings suggest that the OS ethanolic extract demonstrated an improving effect on memory and hence could serve as a potential therapeutic target for the treatment of neurodegenerative diseases like AD.

## Introduction

Neurodegenerative diseases have emerged to become a globally critical burden with the aging population. The number of Alzheimer’s patients have steadily increased over the years with currently more than 46 million people living worldwide with the disease and the number is expected to increase to 131.5 million by 2050 (Mat et al., 2017). Alzheimer’s disease (AD) is the most common cause of cognitive impairment in the elderly population and is characterized by various symptoms that include learning and memory impairment, cognitive dysfunction, language impairment and behavioral dysfunction like depression, agitation and psychosis that continue to become more severe with the disease progression (Brookmeyer et al., 2007; Mahdy et al., 2012). Thus, due to the debilitating nature of the disease, it continues to exist as a huge societal social and economic burden.

One of the key neuropathological features underlying the symptoms associated with Alzheimer’s is neuronal loss and when examined microscopically, the presence of senile plaques and neurofibrillary tangles (NFTs) serves as the main features of the disease. A number of mechanisms have been conjectured to further elucidate the pathogenesis of Alzheimer’s like cholinergic dysfunction, oxidative damage, beta amyloid toxicity, hyperphosphorylation of tau protein, and inflammation of senile plaques (Mahdy et al., 2012; Von Bernhardi et al., 2015). The cholinergic system that comprises of the cholinergic neurotransmitters play a vital role in memory processing whereby loss of the cholinergic neurons and its subsequent decrease results in learning and memory dysfunction characteristic of Alzheimer’s (Watanabe et al., 2009; El-Marasy et al., 2012; Blake et al., 2014). Thus far there are yet to be disease-modifying drugs approved for Alzheimer’s. The medications available are only capable of temporarily alleviating the symptoms of cognitive impairment; however, they do not halt the inevitable progression of the disease. To date, four cholinesterase inhibitors or ChEI (tacrine, rivastigmine, donepezil and galantamine) and a partial NMDA receptor antagonist (memantine) are the only approved treatment options for AD. However, these drugs fail to completely cure the disease, which warrants a search for newer class of targets that would eventually lead to effective drugs for the treatment of AD (Obulesu and Rao, 2011). Thus, major therapeutic research is underway to explore the memory enhancing activities of natural products.

The pharmacological and therapeutic effects of traditional medicinal plants have been associated with the various chemical constituents isolated from their crude extract whereby in particular, active constraints that demonstrate antioxidant activity have been linked to play a central role in various neurodegenerative diseases (Silva et al., 2005; Mahdy et al., 2012). *Orthosiphon stamineus* (OS) Benth. (Lamiaceae) is an Asian folklore medicinal plant that has been employed as treatments for various diseases like influenza, inflammation, urinary tract infections, and angiogenesis related conditions like cancer (Geng et al., 2013; Yehya et al., 2018). OS have been reported to demonstrate anti-inflammatory, antioxidant, antibacterial and hypoglycemic effects (Awale et al., 2003; Akowuah et al., 2004; Ho et al., 2010; Abdelwahab et al., 2011; George et al., 2015). Additionally, several scientific studies have also reported the safety profile of 50% ethanol extract of OS in *in vivo* rat models and the LD_50_ have been said to be more than 5000 mg/kg (Chin et al., 2008; Mohamed et al., 2011; Yehya et al., 2018). Phytochemical studies have demonstrated that OS leaves extracts contain more than 20 phenolic bioactive compounds like rosmarinic acid, 2,3-dicaffeoyltartaric acid, eupatorine, sinesitin, oleanolic acid, ursolic acid, pentacyclic triterpenes, and b-sitosterol (Awale et al., 2003; Shin et al., 2015; Yehya et al., 2018). Among these active compounds, rosmarinic acid has been reported to be the main flavonoid present in the 50% ethanol extract of OS extract and plays a central role for the various pharmacological activities exerted by the OS extract. Flavonoids which are the principal group of polyphenols are also reported to be efficacious in decreasing oxidative stress and are said to promote various physiological benefits, particularly in learning and memory, scavenging free radicals and cognitive impairment (Bhullar and Rupasinghe, 2013; Bhullar and Rupasinghe, 2015; Ghumatkar et al., 2015). Besides that, standardized ethanolic extract of OS were also found to be able to reverse age-related deficits in short-term memory as well as prevent and reduce the rate of neurodegeneration (George et al., 2015).

Additionally, preliminary studies of OS extract have also demonstrated neuroprotective and choline esterase inhibitory effects; this in turn further indicates OS extract’s potential in prompting CNS related reactions. Although OS extract possesses various uses, there are yet no studies on its neuropharmacological activities against AD-like conditions. Therefore, this present study aimed at distinguishing the anti-amnesic potential of this plant derived flavonoid memory deﬁcits in a rat model of cognitive impairment caused by scopolamine.

## Materials and Methods

### Plant Materials

The 50% ethanolic OS extract was procured from NatureCeuticals Sendirian Berhad, Kedah DA, Malaysia. The extract from leaves of OS was prepared under GMP-based environment using DIG-MAZ technology by Natureceuticals Sdn. Bhd., Malaysia. The DIG-MAZ is an extraction system which involves all of the key extraction processes like percolation, digestion, maceration, and distillation. The extract was kept in an airtight container until further experimentations. The OS extract was dissolved in distilled water and filtered using a membrane filter unit (0.22 lm) before being administered to the rats for the study.

### Experimental Animals

In-house-bred adult male Sprague Dawley rats weighing between 200–300 g and between 6 and 8 weeks old were acquired from the animal facility of School of Medicine and Health Sciences of Monash University Malaysia. The rats were kept and maintained in cages under standard husbandry conditions (12:12 h light/dark cycle, at controlled room temperature (22 ± 2°C), stress free, water *ad libitum*, standard diet, and sanitary conditions). Prior to the experiment, the rats were allowed to acclimatize for a period of 1 week to reduce environmental stress. All the experimental protocols were approved and conducted according to the approval of the Monash Animal Research Platform (MARP), Australia with the reference number MARP/2016/028.

### Experimental Design

The range of OS extract doses was determined following the pre-screening results. OS extract, donepezil and scopolamine were prepared by dissolving it in saline. Normal control rats were administered with saline throughout the experiment. The treatments were given both orally and intraperitoneally (i.p.) at a volume corresponding to 0.1 ml/100 g of bodyweight. All experiments were performed in a balanced design (eight animals per group) to avoid being inﬂuenced by order and time. The behavioral studies were divided into two categories namely the nootropic and the scopolamine models.

### Nootropic Model

Group 1: Normal control (saline)
Group 2: Positive control [Donepezil (DPZ) 1 mg/kg]
Group 3: Low dose of OS (50 mg/kg OS)
Group 4: Medium dose of OS (100 mg/kg OS)
Group 5: High dose of OS (200 mg/kg OS)

### Scopolamine Model

Group 1: Normal Control (saline)
Group 2: Positive control (DPZ 1 mg/kg)
Group 3: Negative control (Scopolamine 1 mg/kg)
Group 4: Low dose of OS (50 mg/kg OS + Scopolamine; 1 mg/kg)
Group 5: Medium dose of OS (100 mg/kg OS + Scopolamine; 1 mg/kg)
Group 6: High dose of OS (200 mg/kg OS + Scopolamine; 1 mg/kg)

**Figure 1:**
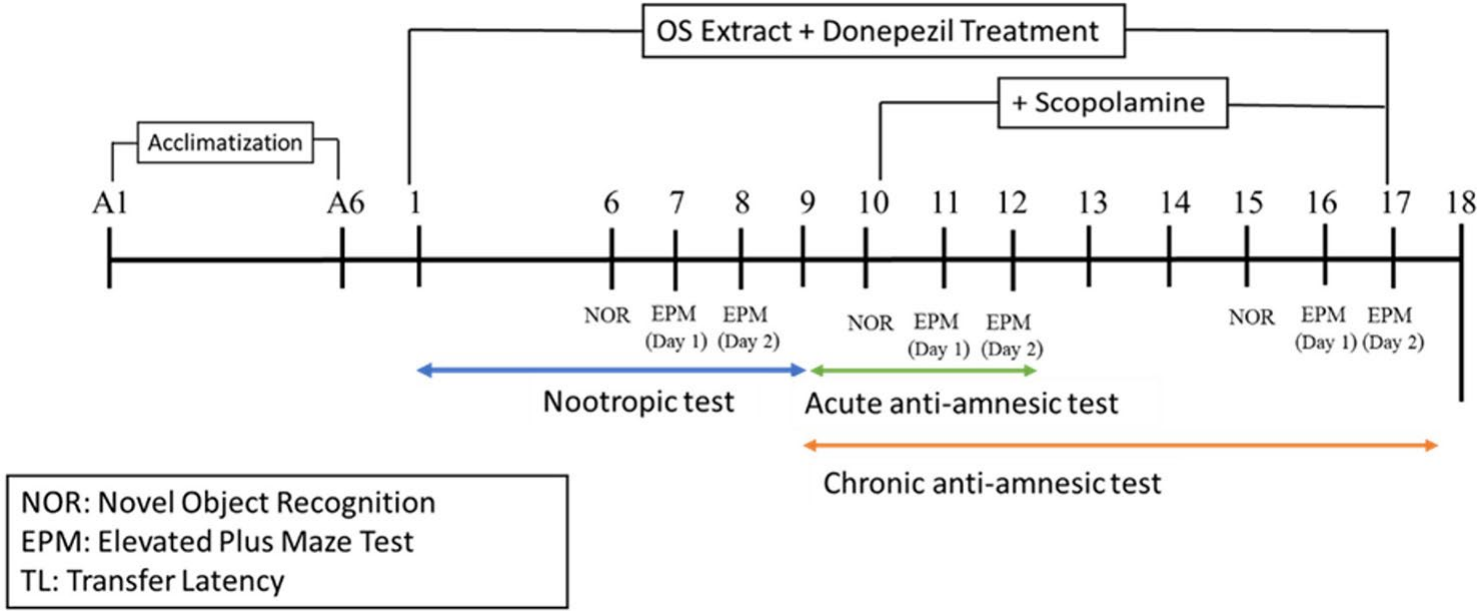
Schematic representation of the experimental procedure.

For the nootropic activity, all the groups received pre-treatment orally for 6 consecutive days before being subjected to a battery of behavioral tests from Day 6 until Day 8 for the novel object recognition (NOR) and the elevated plus maze (EPM) tests, respectively, as observed in **Figure 1**. For the scopolamine model, amnesia was induced in all the groups except the control group by daily intraperitoneal injections of scopolamine (1 mg/kg) for 9 days after OS extract pre-treatment (Day nine to Day 17). Thirty minutes prior to the administration of scopolamine, NOR was conducted on Day 10 and Day 15, and EPM was carried out on Days 11 and 12, and Days 16 and 17 of the study as seen in **Figure 1**. At the end of the experiment, the rats were sacrificed, and their brains were isolated for further biochemical and immunohistochemistry analysis.

### Novel Object Recognition

In the object recognition task, the experimental apparatus consisted of an open field box (40 × 40 × 40 cm) made of black acrylic material. The method used was the same as described by (Ennaceur and Delacour, 1988), with slight modifications. The behavior test was conducted between 9:00 AM and 6:00 PM under dim red-light illumination conditions. The objects to be discriminated were two similar transparent cultured flasks filled with water and a toy Lego of the same height (new object). One day prior to the experiment, each rat was habituated to the open field box without any object for 10 min. On the experiment day, during the first trial, each rat was placed in the open field for 5 min and allowed to freely explore the two identical objects (transparent cultured flasks with water). After 90 min of post-training session, one old object used during the training session was replaced by a novel object and the rat was left to explore the objects for 2 min. The time spent with each object was recorded and evaluated using SMART software version 3.0 (Panlab, Harvard Apparatus). Both objects presented during the test session were different in texture, color, and size. The open field box was cleaned with 70% ethanol between runs to minimize scent trails. The recognition index was computed using the formula [TB/(TA + TB) * 100] where TA and TB are time spent exploring familiar object A and novel object B respectively (Batool et al., 2016). Exploration of an object was deemed when a rat sniffed or touched the object with its nose and/or forepaws.

### Elevated Plus Maze Test

The elevated plus maze test measures anxiety in animals but a significant parameter measured in EPM called the transfer latency (the time taken for the animal to move from an open arm to the closed arm) has been shown to be noticeably reduced if the animal has had prior experience of entering into the open and closed arm and this reduced transfer latency has been demonstrated to be associated with memory process and an increase in inflexion ratio indicates nootropic activity. Additionally, several studies of various nootropics and amnesic agents on EPM have further reiterated this model as a widely accepted paradigm to study learning and memory processes in rodents (Itoh et al., 1990; Yadav et al., 2017). In EPM, transfer latency on Day 1 is deemed as acquisition (learning) and memory retention is then examined after 24 h.

The EPM apparatus consists of four arms of equal dimensions, i.e., two open arms (50 × 10 cm) that are crossed with two closed arms, enclosed by high walls of 40 cm high. These arms are connected with the help of a central square (10 × 10 cm) that gives an appearance of a plus sign to the maze. This maze is elevated from the ground by 50 cm. The method used was the same as described by (Vasudevan and Parle, 2006). The behavior test was conducted between 9:00 AM and 6:00 PM under dim red-light illumination conditions. The memory was assessed in EPM in two sessions, 24 h apart. During the training session, the rats were placed at the end of the open arm, facing away from the central platform. With the help of the stopwatch, the transfer latency (TL1) was noted, i.e., the time taken by rat with all its four legs to move into any one of the enclosed arms. If the rat failed to enter any one of the enclosed arms within 90 s, it was gently pushed into one of the two enclosed arms and the TL was assigned as 90 s. The rat was allowed to explore the maze for the next 10 s and then returned to its home cage. The maze was cleaned with 70% ethanol between runs to minimize scent trails. To assess memory, the retention test phase was carried out 24 h after the training session whereby a decrease in time latency (TL2) during the test session was deemed as an index of memory improvement. The cut-off time for each rat to explore the maze in both the phases (training and test) was 90 s.

The transfer latency was expressed as inflexion ratio, calculated using the formula:

$$\mathrm{IR}=\frac{\left(L1-L0\right)}{L0}$$

L0: Initial TL (s) on the 1st day and

L1: TL (s) on the 2nd day.

### Tissue Processing

All the rats were sacrificed 1 hour after the behavioral test under ketamine-xylazine anesthesia. In each group half the rats (n = 4/group) were fixed with 4% paraformaldehyde (PFA) for immunohistochemistry analysis while the remaining half of the rats (n = 4/group) were used for gene expression analysis. The hippocampal region from the whole brain was isolated immediately and were homogenized on ice cold 200 µL Trizol and stored at −80°C for real-time PCR analysis.

### Gene Expression

Total RNA from the rat brain’s hippocampal region was extracted following the method employed by (Bhuvanendran et al., 2018), with some minor modifications. The single-step method, phenol-chloroform extraction and Trizol reagent (Invitrogen) was used to isolate the total RNA from the hippocampal region. Briefly, the tissues were homogenized in 200 µL of Trizol solution. The mixture was then extracted using chloroform and centrifuged at 135,000 rpm at 4°C. The alcohol was removed, and the pellet was washed twice with 70% ethanol and resuspended in 20 µL of RNase free water. RNA concentration was determined by reading absorbance at 260 nm using Nanodrop. A 500 ng amount of total RNA was reverse transcribed to synthesize cDNA using Quantitect^®^ Reverse Transcription Kit according to the manufacturer’s protocol. Then the mRNA expression of genes encoding cAMP response element-binding protein (CREB1), brain-derived neurotrophic factor (BDNF), tropomyosin receptor kinase B (TrkB), and IMPDH2 in the hippocampus was measured *via* real-time PCR using the StepOne Real-Time PCR system. Subsequently, the cDNA from the reverse transcription reaction was subjected to Real-Time PCR using QuantiNova^™^ SYBR^®^ Green PCR kit according to manufacturer’s protocol. The comparative threshold (C_T_) cycle method was used to normalize the content of the cDNA samples, which consists of the normalization of the number of target gene copies versus the endogenous reference gene, IMPDH2.

### Immunohistochemistry

Immunohistochemical analysis was performed by assessing neurogenesis using Doublecortin (DCX) in the hippocampus. Four brain tissues from each group were immersed in the fixative solution, 4% paraformaldehyde overnight and were methodically cryoprotected in 10%, 20%, and 30% sucrose solution respectively for 24 h. The brains were then embedded in 15% Polyvinypyrrolidone, frozen using dry ice and cut into coronal frozen sections (40 µm) using a Leica CM3050 cryostat. The sections were stored in an anti-freeze buffer. The free-floating sections were subjected to endogenous peroxidase quenching with 1% H_2_O_2_ in methanol for 30 min. After washing with phosphate buffered saline, PBS, the tissues were treated with blocking buffer (1% Bovine Serum Albumin and 0.3% Triton X-100) for 1 hour followed by incubation with primary DCX (1:250, Abcam) antibodies overnight at 4°C. After washing with PBS, the tissues were then biotinylated with goat anti-rabbit secondary antibody (Abcam) for 2 h. The tissues were then subsequently washed with PBS and exposed to an avidin biotin peroxidase complex (Vectastain ABC kit, Vector) for another 2 h. The peroxidase activity was then visualized using a stable diaminobenzidine solution (DAB, Sigma). All immunoreactions were observed under a microscope (BX41, Olympus) and these results were quantified using DigiAcquis 2.0 software.

### Statistical Analysis

Data obtained from all studies were expressed as mean ± SEM. The data were analyzed using one-way analysis of variance (ANOVA) followed by Dunnett *post hoc* test. The P-values of *P < 0.05, **P < 0.01, and ***P < 0.001 were considered as statistically significant. All the experimental groups were compared with the Scopolamine (SCP) 1 mg/kg group except for the nootropic model where the experimental groups were compared with the control group.

## Results

### Scopolamine Induced Model Behavioral Analysis

#### Effect of OS Extract in Nootropic Model

**Figure 2:**
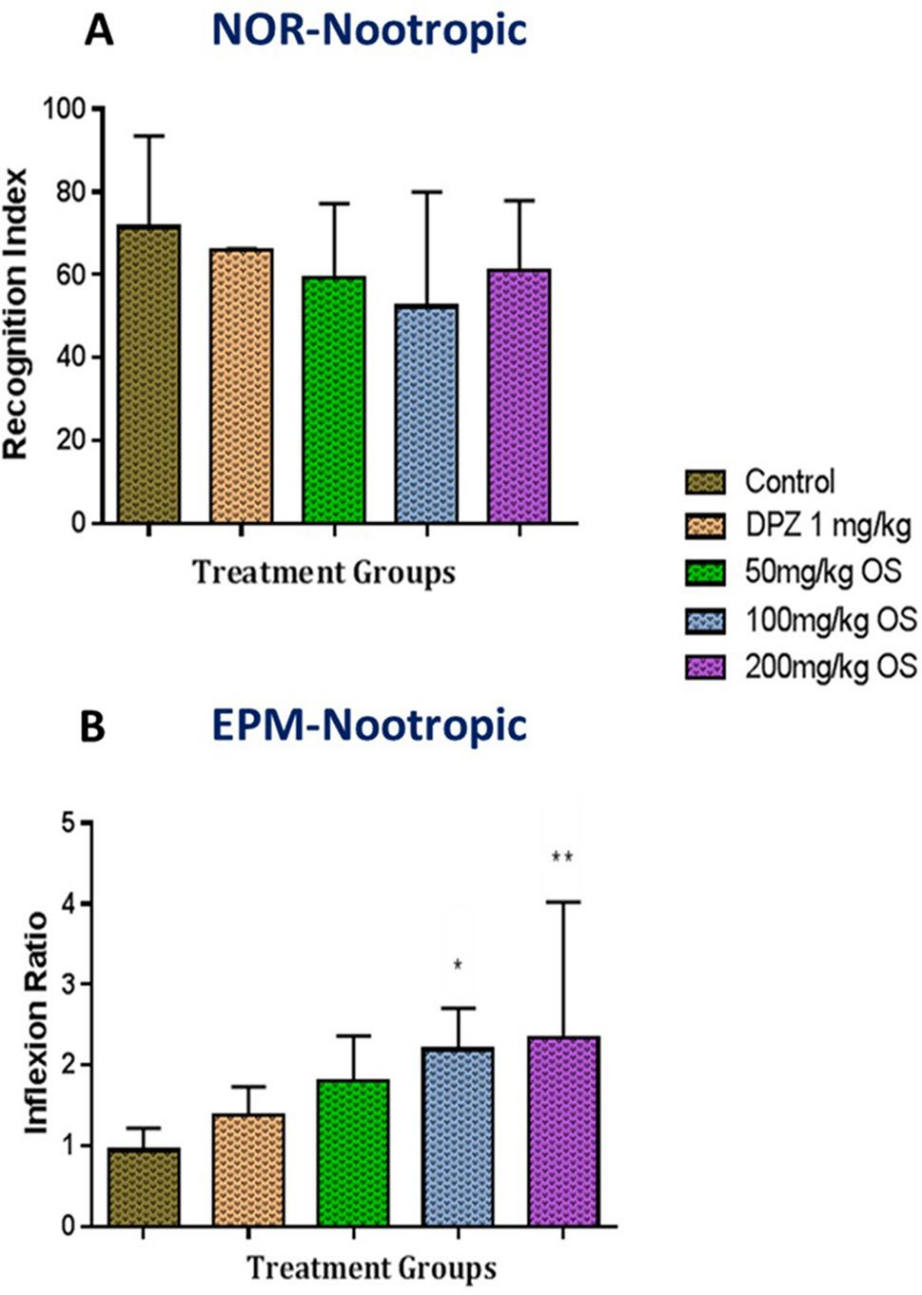
Behavioral analysis for novel object recognition (NOR) and elevated plus maze (EPM). **(A)** represent the graph plot for the recognition indices in NOR for the nootropic model **(B)** represents the graph plot for the inflection ratios in EPM for the nootropic model. The behavioral analysis for **(A**, **B)** were compared to control group. Data are expressed as Mean ± SEM, n = 8 and statistical analysis by one-way ANOVA followed by Dunnett test *P < 0.05 and **P < 0.01.

The NOR test was used to evaluate whether OS treatment could reverse scopolamine-induced recognition impairment whereby the effect of OS extract at different doses were assessed following 7 days of pretreatment. Based on the results obtained in **Figure 2A**, pretreated group of OS extract demonstrated an increase in recognition index [F (4, 32) = 1.096, P = 0.3752] for novel object particularly observable in animals treated with dose of 50 mg/kg and 200 mg/kg OS extract. Overall, it can be said that the preference for the novel object was more or less the same among the OS treated groups when compared to the controls.

The memory function was also assessed using the EPM test to gauge the spatial long-term memory. Based on the results obtained in **Figure 2B**, the time taken for each rat to move from the open arm to either of the enclosed arms on the first trial (familiarization session), termed transfer latency 1, did not significantly differ between groups. However, during the test session, termed transfer latency 2, a decrease in time for transfer latency 2 was observed within the groups. Significant results were observed in rats administered with 100 mg/kg and 200 mg/kg OS extract thus indicating improvement in inflexion ratio [F (4, 35) = 3.713, P = 0.0127] observed among all groups. These results demonstrate that supplementation of OS extract significantly improved memory function in rats thus demonstrating optimal nootropic effects.

#### Effect of OS Extract in Acute Scopolamine Model

**Figure 3:**
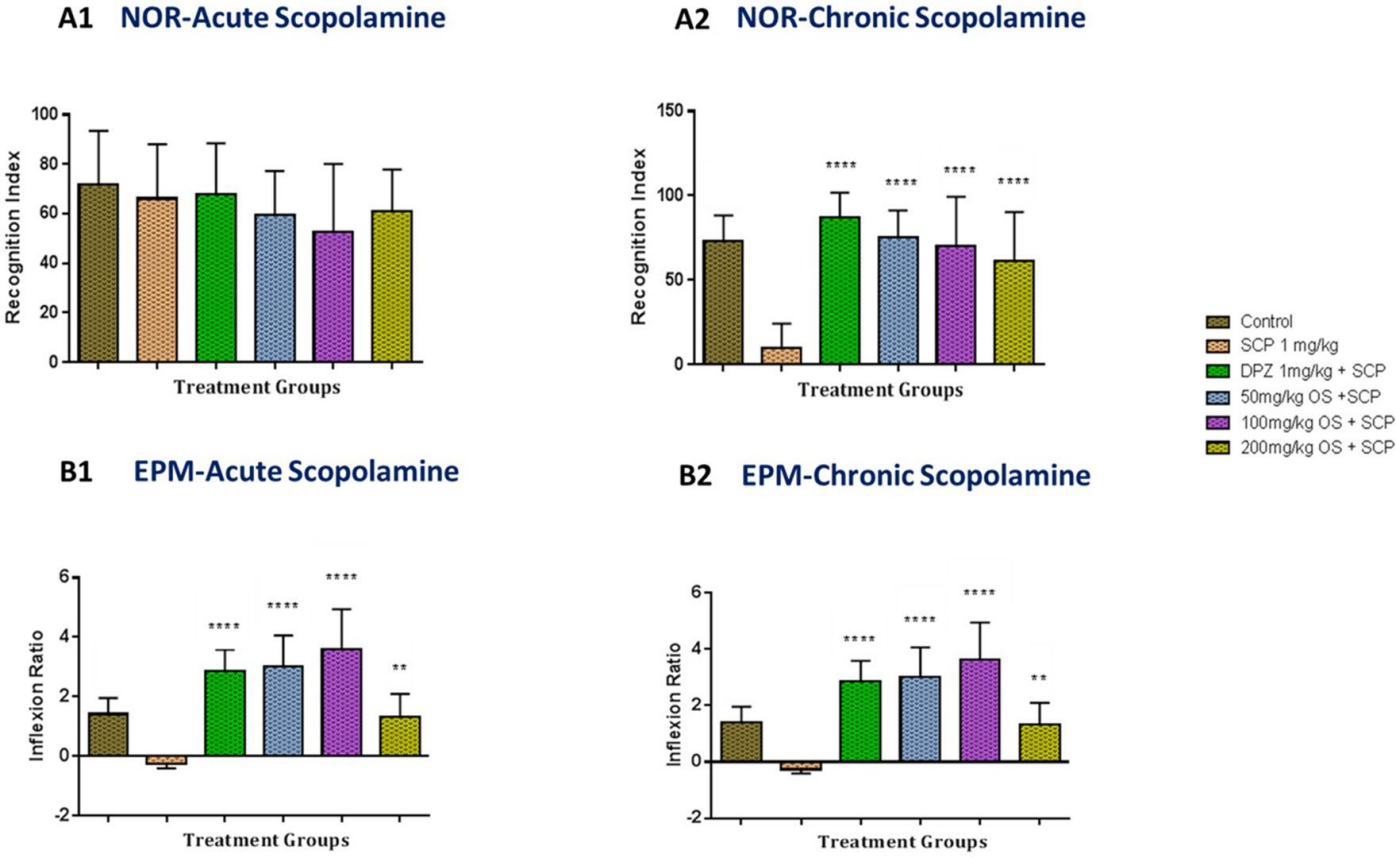
Behavioral analysis for NOR and EPM. **(A1** and **A2)** represents the graph plot for the recognition indices in NOR for both the acute and chronic scopolamine model respectively **(B1** and **B2)** represents the graph plot for inflection ratios in EPM for both the acute and chronic scopolamine model. All the behavioral analysis was compared to the negative control (SCP 1 mg/kg). Data are expressed as Mean ± SEM, n = 8 and statistical analysis by one-way ANOVA followed by Dunnett test **P < 0.01 and ****P < 0.0001.

In the acute scopolamine-induced memory impairment in rats as depicted in **Figure 3 (A1)**, the percentage of recognition index for the positive control (donepezil 1 mg/kg), negative control (scopolamine 1 mg/kg) and the OS extract treated groups, were found to be unchanged, indicating that the acute scopolamine-induced memory was not impaired [F (5, 38) = 0.691, P = 0.6333].

For the EPM test, the scopolamine administered group (negative group) demonstrated a decrease in inflexion ratio when compared to the control and the other treated groups as depicted in **Figure 3 (B1)**. When donepezil (positive group), a well-established standard drug for Alzheimer’s disease was administered, a significant increase in inflexion ratio was observed when compared to the scopolamine treated rats. Similarly, significant improvement in inflexion ratio [F (5, 42) = 23.32, P < 0.0001] was observed in all the 3 doses of OS extract with both 50 mg/kg and 100 mg/kg demonstrating a notable increase in inflexion ratio when compared to the scopolamine treated group indicating that the memory impairment induced by scopolamine was reversed. These results further reiterate that OS extract were able to improve retention memory.

#### Effect of OS Extract in Chronic Scopolamine Model

In the chronic scopolamine model, the NOR test showed that there was a relatively large drop in the percentage of recognition index for the scopolamine treated group as shown in **Figure 3 (A2)**. The percentage of recognition index for all the OS extract groups were observed to have a significant increase [F (5, 42) = 13.74, P < 0.0001] when compared to the scopolamine treated group indicating improved memory retention.

For the EPM test as observed in **Figure 3 (B2)**, when chronic exposure of scopolamine was given to the rats, a notable decrease in inflexion ratio was observed in the scopolamine treated group whereas both 50 mg/kg and 100 mg/kg dose of OS extract demonstrated a significant improved inflexion ratio [F (5, 42) = 23.32, P < 0.0001] when compared to the scopolamine treated group indicating that the increase could be due to the repeated exposure of scopolamine. However, the 200 mg/kg OS extract demonstrated a significant increase in inflexion ratio when compared to the negative group, but when compared between the OS extract doses, it was much lower compared to the other two doses. Based on these results, we can say that OS extract does improved memory retention when exposed repeatedly to scopolamine.

### Scopolamine Induced Model Gene Expression Analysis

#### Gene Expression in the Hippocampal Region

**Figure 4:**
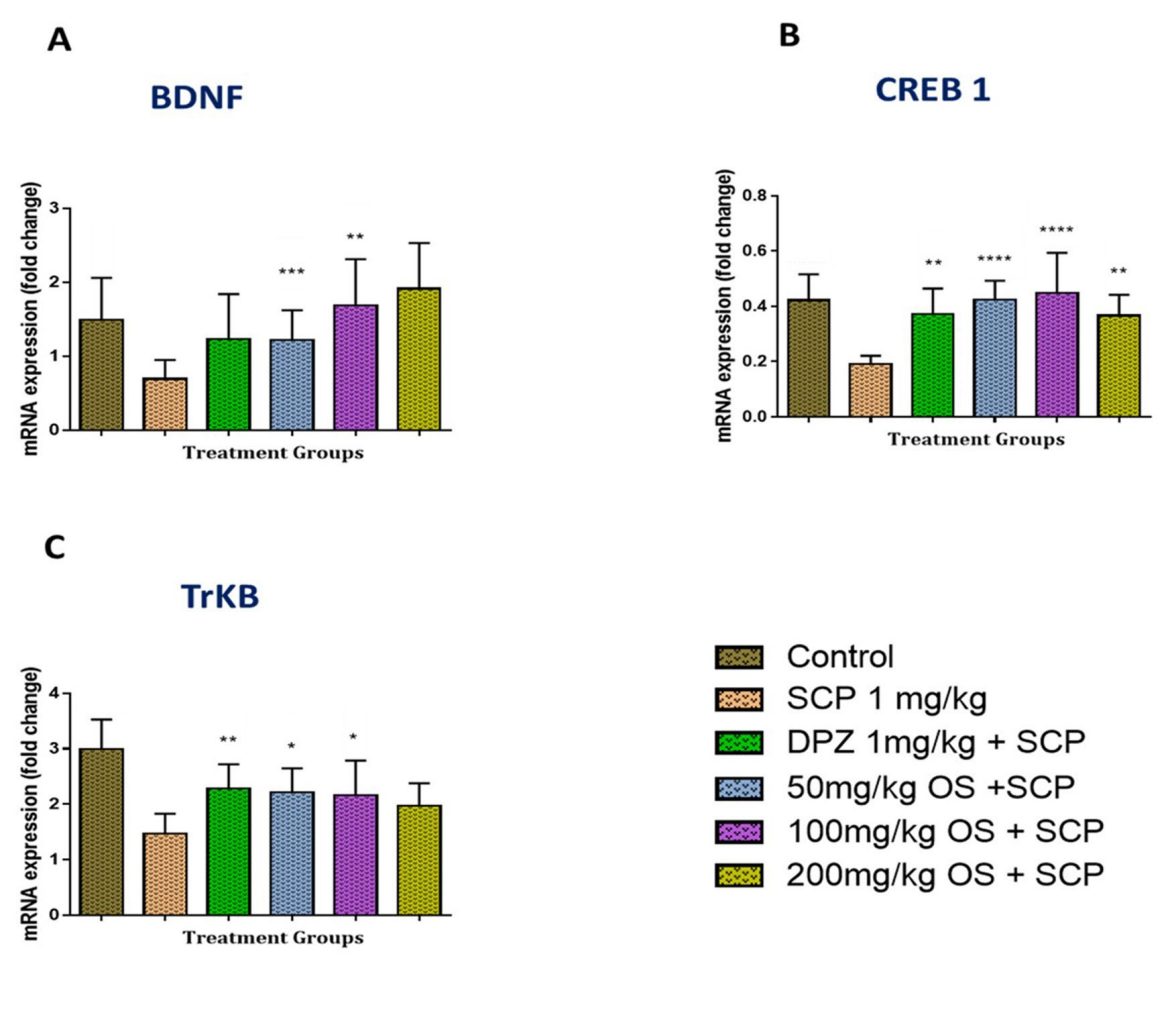
Gene expression in the rat hippocampi determined by real time-PCR. The genes included are **(A)** BDNF, **(B)** CREB1, and **(C)** TrKB. All changes in the expression levels were compared to the negative control group (SCP 1 mg/kg). Data are expressed as Mean ± SEM, n = 4 and statistical analysis by one-way ANOVA followed by Dunnett test *P < 0.05, **P < 0.01, ***P < 0.001, and ****P < 0.0001.

In the hippocampal region, the BNDF mRNA levels were found to be significantly down regulated [F (6, 49) = 3.948, P = 0.0027] when injected with scopolamine as compared to the control group, as depicted in **Figure 4A**. Similarly, even the CREB1 [F (6, 49) = 7.517, P < 0.0001] and TRKB [F (6, 49) = 10.23, P < 0.0001] mRNA levels were observed to be down regulated when given scopolamine as shown in **Figures 4B, C**, respectively. This down regulation was ameliorated significantly by OS extract pretreatment as compared with the negative (scopolamine 1 mg/kg) group. Moreover, in all the three mRNA expression levels, namely CREB1, BDNF, and TRKB were observed to be significantly higher when the rats were treated with 100 mg/kg OS extract. Similar up regulation in the mRNA levels were also observed in the positive group rats that were treated with donepezil.

#### Scopolamine-Induced Model Immunohistochemistry Analysis

**Figure 5:**
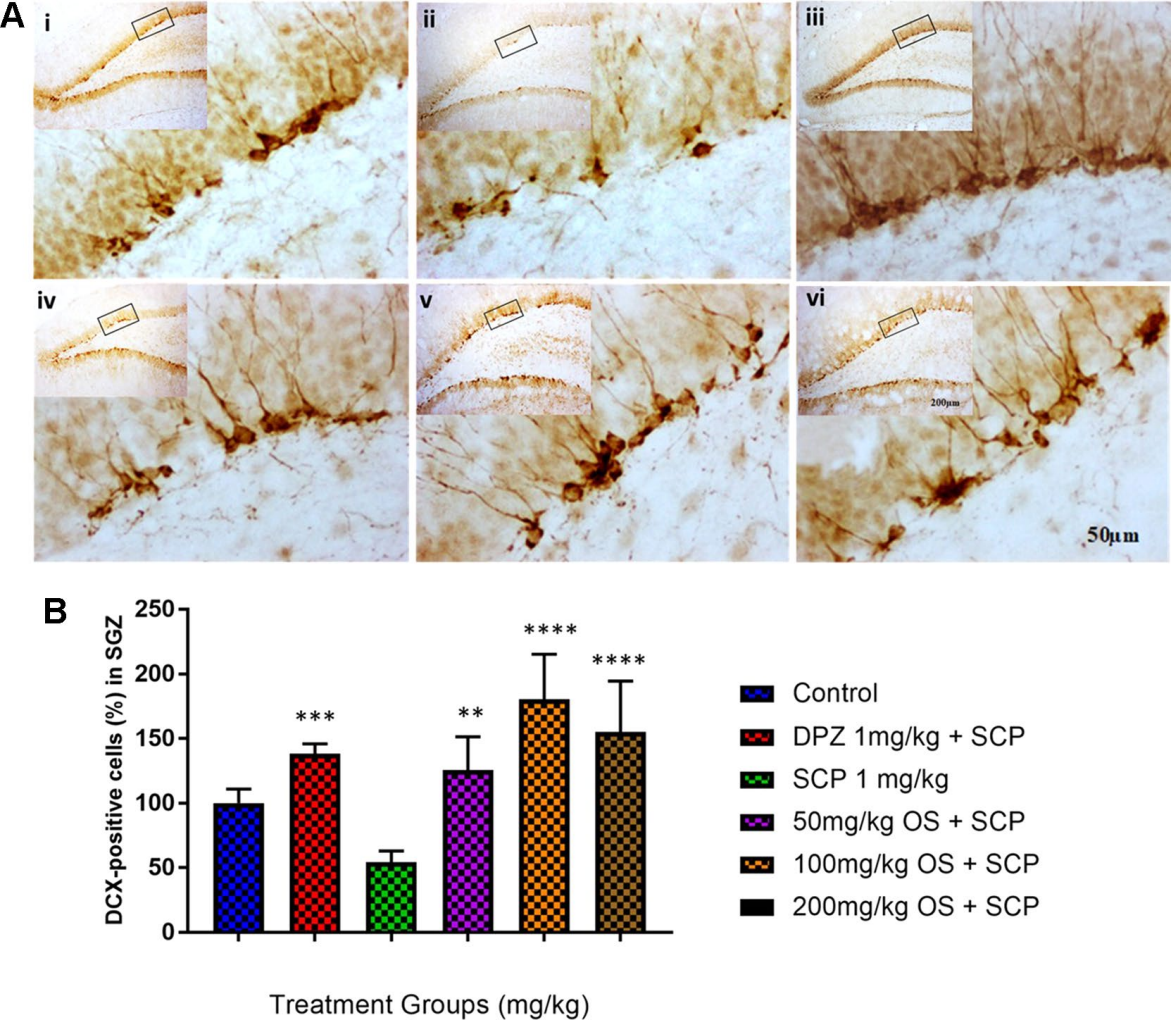
DCX immunohistochemical analysis of the effects of OS extract in improving scopolamine-induced suppression of neurogenesis in the dentate gyrus. **(A)** DCX-positive staining in immature neurons is shown in the SGZ of the dentate gyrus. Photomicrographs of the hippocampal section of treatment groups was **(i)** Control **(ii)** SCP 1 mg/kg alone **(iii)** DPZ 1 mg/kg + SCP 1 mg/kg **(iv)** 50 mg/kg OS + SCP 1 mg/kg **(v)** 100 mg/kg + SCP 1 mg/kg **(vi)** 200 mg/kg + SCP 1 mg/kg. Representative photomicrographs were taken at magnifications of 40× and 200×. **(B)** Quantification of DCX population. Data are expressed as means Mean ± SEM, n = 4 – 6 and statistical analysis by one-way ANOVA followed by Dunnett test **P < 0.01, ***P < 0.001 and ****P < 0.0001.

In the immunohistochemical studies, scopolamine injection was observed to have suppressed adult neurogenesis, shown as distributed dendrites and neuron bodies in the dentate gyrus (DG) region by DCX staining, particularly in the sub-granular zone (SGZ). Additionally, pretreatment with OS extract were observed to have ameliorated [F (5,18) = 12.74, P < 0.0001] the adult neurogenesis by enhancing immature neurons in the SGZ compared to the scopolamine treated group, as depicted in **Figure 5**.

## Discussion

The present study demonstrated that pretreatment with OS extract improved memory retention as evident by the improved inflexion ratio observed in the EPM test as well as the increase in the recognition index observed in the OS treated rats. Previous studies have demonstrated scopolamine to show profound amnesic effects in various learning paradigms through the disruption of the cholinergic neurotransmission whereby when given acutely, scopolamine was said to produce spatial memory deficit (Goverdhan et al., 2012; Ghumatkar et al., 2015). In our study, similar results were observed whereby the scopolamine treated group in both the acute and chronic model for the EPM test demonstrated decreased inflexion ratio indicating impairment of spatial memory. Similarly, in the NOR test, the recognition index was decreased in the scopolamine treated group in the chronic model indicating cognitive deficit. However similar results were not observed in the acute model further corroborating that the NOR test were not influenced by the acute scopolamine treatment. Donepezil is a well-established drug used to treat dementia associated with AD (Sumanth et al., 2010; Sumanth et al., 2010) and was hence used as the positive control in our *in vivo* study as it was said to be able to reverse scopolamine induced memory impairment in previous studies (Cachard-Chastel et al., 2008; Ghumatkar et al., 2015). The rats that were treated with 50 mg/kg OS extract showed a decrease in transfer latency in which the rats were able to remember and enter the closed arm quickly compared to the training session, which was observable by the improved inflexion ratio, however the rats that were treated with 200 mg/kg did show improved memory retention as compared to the scopolamine treated group but not as that observed in the 50 mg/kg OS treated group. A ceiling effect was observed with higher doses. Therefore, based on the behavioral analyses, we can conclude that the spatial memory was improved in both the acute and chronic scopolamine model and this improved performance may be attributed to its enhanced cholinergic neuronal transmission.

Scopolamine is a non-selective muscarinic cholinergic receptor antagonist that inhibits the central cholinergic neuronal activity which in turn leads to impairment in spatial learning and memory in rodents and humans (Konar et al., 2011). The central cholinergic system is also found to be closely associated with neurogenesis and/or cell proliferation in the hippocampus (Yoo et al., 2011). In this study, we illustrated the nootropic and neuroprotective effects of OS extract in a scopolamine induced amnesia model. The medicinal value of OS extract has been well recognized, particularly in regard to its anti-oxidant and anti-inflammatory activities (Arafat et al., 2008) In particular, rosmarinic acid which is the main flavonoid component of OS extract has demonstrated various pharmacological properties that may potentially hinder neurodegeneration and improve memory and cognitive functioning (Essa et al., 2012). Thus, this cocktail of flavonoids could be in turn responsible for the positive behavioral results observed.

The underlying mechanism for the improvement in memory retention observed in the behavioral studies was further explored by evaluating the biochemical parameters like expression of CREB1, BDNF. and TrkB genes in rats treated with OS extract and scopolamine. Adult hippocampal neurogenesis and neuroplasticity are modulated by many neurotrophic factors such as BDNF (Begni et al., 2017; Begni et al., 2017). BDNF is a small dimeric protein which is one of the neurotrophic factors that play a vital role in regulating not only the neuronal development, maintenance and survival, but also in the cognition, formation and storage of memory. In 1991, reduced expression of BDNF were first seen in hippocampus samples from AD donors suggesting that this decrease may contribute to the progressive cell death characteristic of AD (Phillips et al., 1991). Furthermore, BDNF was also found to promote the survival of all major types of neurons related to functional changes in AD, and has been suggested as an essential contributor of the etiology of neurodegenerative disorders (Schindowski et al., 2008). As stated earlier, BDNF is involved in neuronal survival and plasticity that binds to high-affinity receptors, TrkB (tropomyosin receptor kinase B) (Givalois et al., 2004). Previous studies have also demonstrated that both BDNF and TrkB play a critical role in long-term synaptic plasticity in the adult brain (Schinder and Poo, 2000; Dwivedi, 2009). BDNF-TrkB interaction promotes the survival and differentiation of neurons and synaptic plasticity of the central nervous systems (Lu et al., 2008; Kim et al., 2010). Thus, a decrease in BDNF and its receptor, TrkB may lead to synaptic and cellular loss and memory deficits characteristic of AD. In the present study, the induction of scopolamine-induced amnesia showed suppression of BDNF and TrkB expressions in the hippocampus. Similar results were also observed in the prefrontal cortex whereby scopolamine reduced mRNAs of BDNF and TrkB. OS extract was found to have increased both the BDNF and TrkB stained cells in the hippocampus and the prefrontal cortex region. In the hippocampus, all the OS extract doses were found to be effective and showed maximum protection by increasing the BDNF and TrkB levels.

On the other hand, CREB1 is a co-factor of CREB and is essential for memory and synaptic plasticity in the central nervous system whereby disruption of phosphorylated CREB within the hippocampal region triggers the progression of neurodegenerative diseases like AD, Parkinson’s disease and Huntington’s disease (Lee et al., 2015). Previous studies have demonstrated that the activation of CREB ameliorated cognitive impairment *via* the cholinergic system (Kotani et al., 2006; Lee et al., 2015). This was congruent with our results whereby the expression of the CREB1 gene was reduced by scopolamine and pretreatment with OS extract markedly increased the CREB1 mRNA levels. So, it can be suggested that OS extract could be a potent treatment for neurodegenerative diseases and its possible mechanism might be modulating the cholinergic activity *via* the CREB-BDNF pathway.

The hippocampus is a pivotal region of the brain that is critical for learning and memory function and is highly susceptible to neuronal injury produced by scopolamine-induced cholinergic activity dysregulation, which can in turn trigger impairment of synaptic plasticity and loss of spatial learning memory (Mattson et al., 2002; Heo et al., 2014a). DCX is a marker of neuroblasts, neuronal precursor cells, and immature neurons. It is associated with structural plasticity in the adult mammalian brain, and has been used as a marker of newly formed neurons in the DG of the adult hippocampus (Bonfanti 2006; Heo et al., 2014b; Heo et al., 2014b). DCX is involved in neuronal migration and development, and it is continuously expressed during adult neurogenesis thus enabling it to be used to measure neurogenesis (Knoth et al., 2010; Heo et al., 2014b). Previous studies have reported decreased DCX expression during aging and thus decrease in neurogenesis (Brown et al., 2003; Hwang et al., 2008; Heo et al., 2014b). In our study, similar results were observed whereby the number of DCX-positive cells in the hippocampal DG was decreased in scopolamine induced rats, whereas the OS treated rats were observed to have increased number of DCX-positive cells. However, further research is necessary to verify its mechanism. Based on the behavior results for EPM, improved inflexion index was observed for both 50 and 100 mg/kg which was equivalent to the positive group, Donepezil indicating that OS at these doses were able to completely reverse the scopolamine induced memory impairment. This was further reiterated by the increase in dendrites and neuron bodies observed. For the 200 mg/kg dose, behavioral studies did show a slight improvement in inflexion index compared to the only scopolamine induced group but when compared to the positive group, Donepezil and the other 2 doses, 50 and 100 mg/kg groups, the improvement was not that convincing. This result was further supported as there were dendrites and neuron bodies observed during the cell counting but not as much as the other 2 doses.

## Conclusion

In conclusion, the present work demonstrated that OS extract was able to revert the scopolamine induced amnesia in the rats thus further distinguishing its anti-amnesic effects. Additionally, we also established that the positive effects of OS extract could be mediated *via* the BDNF-TrKB pathway, CREB-BDNF pathway and also the hippocampal neurogenesis. This suggests that the OS extract could be a promising candidate as a memory enhancer or as a therapeutic treatment for neurodegenerative diseases like AD.

## Ethics Statement

All the experiments were conducted according to the approved protocols by the Monash Animal Research Platform (MARP) animal ethics committee in Australia.

## Author Contributions

TR performed all the experiments and was responsible for the writing of the manuscript in its entirety. YK helped in designing of gene expression and immunohistochemistry study. MS and IO were involved in conceptualizing, designing of the study, result analysis, and manuscript editing. All authors gave their final approval for the submission of the manuscript.

## Funding

Authors are thankful to NKEA Research Grant Scheme (NRGS), Ministry of Agriculture and Agro-Based Industry Malaysia (Grant No. NH1014D066) for providing funding support.

## Conflict of Interest

The authors declare that the research was conducted in the absence of any commercial or financial relationships that could be construed as a potential conflict of interest.
